# A history of maternal separation drives systemic aging-associated signatures in middle-aged male rats

**DOI:** 10.3389/fncel.2026.1809602

**Published:** 2026-05-04

**Authors:** Pratik R. Chaudhari, Arvindkumar H. Chaurasiya, Ashok D. B. Vaidya, Mahesh J. Kulkarni, Vidita A. Vaidya

**Affiliations:** 1 Department of Biological Sciences, Tata Institute of Fundamental Research, Mumbai, India; 2 Biochemical Sciences Division, CSIR-National Chemical Laboratory, Pune, India; 3 Kasturba Integrative Health Sciences-Medical Research Foundation, Mumbai, India

**Keywords:** advanced glycation end-products, aging, early stress, mass spectrometry, methylglyoxal, oxidative stress

## Abstract

Early adversity programs induce changes that can accelerate biological aging. Using the early stress model of maternal separation (MS), we assessed oxidative, metabolic, and biochemical consequences in serum derived from middle-aged male rats to investigate systemic correlates of physiological aging in MS animals. We noted significant increases in serum corticosterone in middle-aged MS male rats, accompanied by reduced serum levels of trophic factors, brain-derived neurotrophic factor (BDNF), and insulin-like growth factor-1 (IGF1). We also found increased oxidative stress markers, such as oxidized low-density lipoprotein (Ox-LDL) in MS animals, concomitant with reduced antioxidant enzyme activity of superoxide dismutase (SOD) and catalase. The serum lipid profile analysis revealed metabolic dysregulation with increased triglyceride, total cholesterol, and LDL levels. Furthermore, mass spectrometric analysis indicated a significant increase in advanced glycation end-product (AGE) modified serum albumin peptides in middle-aged MS male rats, accompanied by enhanced expression of the precursor for AGEs, methylglyoxal (MGO), and the soluble form of the receptor for AGEs (sRAGE). Collectively, these findings suggest that the early stress of MS evokes long-lasting systemic changes that persist into middle age and reflect glyco-oxidative damage, dyslipidemia, disrupted trophic factor signaling, and enhanced accumulation of AGEs, which could contribute mechanistically to cellular and physiological aging processes.

## 1 Introduction

Disruption of early-life environments can exert profound and enduring influences on physiology, shaping health trajectories later in life (Anisman et al., 1998; Barker, 2007; Bale et al., 2010). Adverse experiences during this critical period are increasingly recognized as key determinants of increased vulnerability to psychopathology and an accelerated aging trajectory (Nelson et al., 2020; Birnie and Baram, 2025; Lemus-Roldan et al., 2025). Epidemiological studies consistently demonstrate that early adversity is associated with increased risk for metabolic dysfunction, cardiovascular disease, neuropsychiatric disorders, and cognitive decline later in life, with some reports documenting increased risk of early mortality (Brown et al., 2009; Keator et al., 2024; Venn et al., 2025). However, the biological mechanisms through which early stress becomes embedded to influence aging processes remain unclear. Notably, numerous epidemiological studies have documented a comorbidity between certain psychiatric disorders and metabolic dysfunctions (Gómez-Ilescas and Silveira, 2025; Silveira et al., 2025). One prominent hypothesis posits that early stress programs long-term dysregulation of oxidative and metabolic homeostasis, resulting in altered systemic aging. Oxidative burden, characterized by heightened oxidative stress concurrent with diminished antioxidant defenses, is considered one of the key contributors to aging and age-related pathologies (Korovesis et al., 2023; Płóciniczak et al., 2025). Sustained oxidative burden evokes distinct modifications on proteins, lipids, and nucleic acids, impairing their normal physiological functions and thus leading to cellular dysfunction and enhanced inflammaging (Krishnamurthy et al., 2024; Płóciniczak et al., 2025). Likewise, metabolic alterations further exacerbate oxidative damage and are closely linked to the accumulation of advanced glycation end-products (AGEs), their precursor methylglyoxal (MGO), and the receptor for AGEs (RAGE). These are potent mediators of glyco-oxidative stress and inflammatory signaling, and their increased burden is a hallmark of aging and metabolic diseases (Chaudhuri et al., 2018; Kold-Christensen and Johannsen, 2020).

Rodent models offer a powerful platform to mechanistically dissect how early adversity shapes biological aging processes (Molet et al., 2014; Chaudhari et al., 2022). MS, a well-established model of early stress, recapitulates aspects of neglect and disrupted caregiving, leading to long-lasting alterations in neuroendocrine, metabolic, and immune function (Rincel and Darnaudéry, 2020; Čater and Majdič, 2022; Zhang et al., 2025). While the impact of MS on behavior and stress reactivity has been extensively characterized, its consequences on systemic aging-related processes in later life remain less well explored. In this context, the present study investigates the long-term impact of MS on systemic signatures in middle-aged rodents, an epoch of life at which early vulnerabilities may manifest as altered aging phenotypes. We focused on the systemic glyco-oxidative stress markers, including AGEs, methylglyoxal (MGO), soluble form of the receptor for AGEs (sRAGE), and oxidized low-density lipoprotein (Ox-LDL), alongside circulating indicators of oxidative defense, metabolic regulation, and trophic factors. Our study also highlights the influence of early stress history on specific markers of glyco-oxidative damage, metabolic dysregulation, and altered trophic factor signaling in middle-aged life, thereby strengthening our understanding of the impact of early stress on aging trajectories.

## 2 Materials and methods

### 2.1 Animals

Sprague–Dawley (SD) rats (*Rattus norvegicus*) were bred in the Tata Institute of Fundamental Research animal facility, housed in groups, and maintained under a 12:12-h light–dark cycle with access to food and water *ad libitum*. All animal procedures were in accordance with the Committee for Control and Supervision of Experiments on Animals (CCSEA) guidelines and were approved by the Institutional Animal Ethics Committee (TIFR/IAEC/2019–4).

### 2.2 Maternal separation paradigm

Animals were subjected to the early stress of maternal separation (MS) from postnatal day (P)2 to P14. Litters born to pregnant primiparous dams were randomly assigned to control (C) or MS groups on P1, with litter size maintained at 10 pups. Pups in the MS group were separated as an entire litter from their dams for 3 h daily (10:00 to 13:00) from P2 to P14, and placed in beakers with bedding and nesting material from their home cage with temperature-controlled heating pads to maintain euthermic conditions. Control litters and dams were left undisturbed in their home cage, except for routine animal facility rearing that involved brief handling during cage maintenance. Each experimental group used in this study had animals derived from 3 to 4 litters per group to minimize any litter-based effects. To avoid the risk of observing litter-specific effects, pups from multiple litters (3–4 per group) were weaned at P28, randomly assigned and housed in sex-matched groups of 3–4 animals per cage. Subsequent experiments were performed in middle-aged (13–14 months) control or MS male rats.

### 2.3 Immunoassays

Middle-aged male control and MS rats (13–14 months) were sacrificed between 9:30 and 11:00 h by rapid decapitation, and trunk blood was collected for biochemical measurements (in both the C and MS groups, *n* = 8). After blood coagulation at room temperature for 30 min, blood samples were centrifuged at 1200 *g* for 18 min in a refrigerated centrifuge (Eppendorf, Germany). Serum collected was stored at −80 °C and subsequently used for immunoassays. The lipid profile was performed at the Shahbazker’s Diagnostic Center, Mumbai, India. Animals were not subjected to fasting and had *ad libitum* access to food and water before blood collection for lipid measurements. The following methods were used for profiling lipids: GPO-phenol aminophenazone (PAP; triglycerides), cholesterol oxidase-phenol aminophenazone (CHOD-PAP; cholesterol), and direct estimation (high-density lipoprotein, HDL; and low-density lipoprotein, LDL). Circulating levels of markers associated with inflammation, oxidative defense, metabolic regulation, and neurotrophic factors were determined using commercially available immunoassay kits in accordance with the manufacturer’s instructions. The following immunoassays were performed on serum derived from control and MS animals in middle-aged life to detect levels of corticosterone (Cort; R & D systems, USA, #KGE009), methylglyoxal (MGO; Cell biolabs, USA, #STA-811), receptor for advanced glycation end-products (RAGE; Abcam, UK, #ab202409), oxidized low-density lipoprotein (Ox-LDL; My bio source inc., USA, #MBS262297), vitamin C (My bio source inc., USA, #MBS721134), brain-derived neurotrophic factor (BDNF; Biosensis, Australia, #BEK2211), and insulin-like growth factor-1 (IGF1; R & D systems, USA, #MG100).

### 2.4 Enzyme activity analysis

Serum harvested from middle-aged control and MS male rats was used to assess the antioxidant enzyme activity (both C and MS, *n* = 8). Activity of superoxide dismutase enzymes, including Cu/Zn (SOD1), Mn (SOD2), and Fe-SOD, was determined using a colorimetric activity kit as per the manufacturer’s guidelines (SOD; Invitrogen, USA, #EIASODC). To quantitatively measure catalase activity in serum samples, a colorimetric kit for catalase was used as per the manufacturer’s guidelines (Invitrogen, USA, #EIACATC).

### 2.5 Liquid chromatography–mass spectrometry (LC–MS) analysis

Serum samples from three individual rats were pooled per sample for LC–MS analysis to obtain sufficient serum material (*n* = 3 per group; each *n* pooled from three rats, and each *n* was run in three technical replicates). Serum protein concentrations from the C and MS cohorts were estimated using the Bradford method, and serum proteins (50 μg) were suspended in urea (8 M)/Tris–HCl (50 mM) at pH 8.0. Proteins were reduced in dithiothreitol (5 mM) at 37 °C for 1 h, followed by alkylation of sulfhydryl groups of cysteine residues in iodoacetamide (15 mM) in the dark under room temperature for 30 min. Trypsin (2 μg) was added to each reaction mixture and incubated overnight at 37 °C for digestion. The digestion reaction was stopped by adding formic acid, and tryptic peptides were desalted using the Sep-Pak C18 cartridges (Waters Corporation, USA), then dried with a vacuum concentrator and stored at −80 °C. The Sequential Window Acquisition of all Theoretical Mass Spectra (SWATH)–Mass spectrometric analysis was performed on a Triple TOF 5600 mass spectrometer (SCIEX, USA) connected to an Ekspert MicroLC 200 (Eksigent, USA). The concentration of reconstituted tryptic peptides was measured at 205 nm using a NanoDrop 2000 spectrophotometer (Thermofisher Scientific, USA). The peptides were separated using the Eksigent C18-RP HPLC column (length 100 mm, ID 0.3 mm, particle size 3 *μ*m, and pore size 120 Å) at 40 °C using a gradient of mobile phase A (water with 0.1% formic acid) and mobile phase B (acetonitrile with 0.1% formic acid) at 7 μL/min flow rate. For peptide separation, the concentration of mobile phase B was increased linearly from 3 to 10% in 20 min, followed by 10 to 30% in 70 min. The mass spectrometric survey scan was performed over a mass scan range of 350–1,250 Da with an accumulation time of 250 ms. The tandem mass spectrometry scan was performed over the mass range of 100–1,600 Da for an accumulation time of 100 ms. The SWATH–mass spectrometry runs were performed in technical triplicate with peptide (1 μg) loading onto the column. Mass spectrometric scans were performed over the mass range 400–1,250 Da, divided into 34 windows of 25 Da each, with an overlap of 1 Da for an accumulation time of 100 ms, whereas tandem mass spectrometry scans were performed at 100–2000 Da under the accumulation time of 70 min. The raw mass spectrometric data of all SWATH-MS runs have been deposited using the ProteomeXchange Consortium through the PRIDE (Perez-Riverol et al., 2025) partner repository with the dataset identifier PXD074495.

### 2.6 Quantification of glycated albumin peptides

Skyline (Version 22.2.0.255, MacCoss Lab) was used to quantify glycated albumin peptides using SWATH–mass spectrometry. The FASTA file of rat serum albumin was used for generating the reference mass spectral library. For data analysis, trypsin was specified as a digestion enzyme. Carbamidomethylation at cysteine (57.021464 Da) was specified as a static modification, whereas Argpyrimidine (Argpyr) at arginine (80.0261 Da), methylglyoxal-derived hydroimidazolone isomer 1 (MG-H1) at arginine (54.010565 Da), N_ɛ_-carboxyethyl-lysine (CEL) modification at lysine (72.021126 Da), and N_ɛ_-carboxymethyl-lysine (CML) modification at lysine (58.0054779 Da) were specified as variable modifications. Precursor ion charge was specified as 2 and 3, whereas fragment ion charge was specified as 1 and 2. The acquisition method was specified as DIA, the product mass analyzer as TOF, the isolation scheme as SWATH (25 m/z), and the resolving power as 30,000. Only tryptic peptides detected with any type of glycation in all samples were selected for quantification, and their retention times were manually corrected as needed. The intense *b* and *y* fragment ions of any precursor consistently detected in all mass spectrometric runs were selected (Supplementary Table 1), and their area under the curve was integrated to obtain the peak area of that particular precursor ion (Supplementary Table 2). The peak areas of unmodified and corresponding glycated albumin precursor ions were exported, and the peak areas of the glycated precursor ions were normalized using the peak area of corresponding unmodified precursor ions.

### 2.7 Novel object recognition task

Control and MS male rats were subjected to the novel object recognition (NOR) task to assess object recognition memory in middle-aged (13–14 months) life (both C and MS, *n* = 8). Animals were habituated to a novel arena (40 cm × 60 cm × 29 cm) for 3 days, 20 min daily. On day 4, animals were allowed to explore two identical objects in the arena for 15 min. Object recognition memory was tested on day 5 by replacing one of the identical objects with a novel object of similar dimensions. An automated tracking system (Noldus Ethovision 3.1, Noldus Information Technology, Netherlands) was used to determine cognitive performance on the NOR task. Discrimination index {[(time spent exploring the novel object - time spent exploring the familiar object)/total object exploration time] X 100} was calculated to assess object recognition memory over a 5-min exploration period. Four days post-NOR task, middle-aged control and MS animals were sacrificed to harvest serum, which was further used to assess circulating levels of Cort and MGO as described in Section 2.3. These biochemical measures were then integrated with the behavioral data obtained from the NOR task to perform cross-correlation analyses, enabling assessment of the relationship between object recognition memory performance and peripheral circulating markers, namely Cort and MGO.

### 2.8 Data representation and statistical analysis

All datasets were tested for normality using the Kolmogorov–Smirnov test on GraphPad Prism 10 (Graphpad Software Inc., USA) before performing appropriate statistical analysis. For statistical analysis on experiments with two groups that followed a normal distribution, a two-tailed, unpaired Student’s *t*-test was performed. To address concerns related to multiple biochemical comparisons, false discovery rate (FDR) correction was applied to the serum biomarker analyses using the Benjamini–Hochberg method, and adjusted values were also reported as FDR-corrected *p* values (Supplementary Table 3). Data are expressed as mean ± standard error of the mean (SEM), and statistical significance was determined at *p* < 0.05. Graphs were generated using the GraphPad Prism 10 software. The number of dots in a graph indicates the number of animals used in the experiment. Both the correlation matrices and Pearson’s correlation coefficient (*r*) were evaluated using GraphPad Prism 10 software. The details of statistical analyses are provided in the supporting information (Supplementary Table 3). The schematic representations were created using BioRender.com.

## 3 Results

### 3.1 Middle-aged MS rats exhibit altered serum markers of stress, glyco-oxidative damage, and growth factors

**Figure 1:**
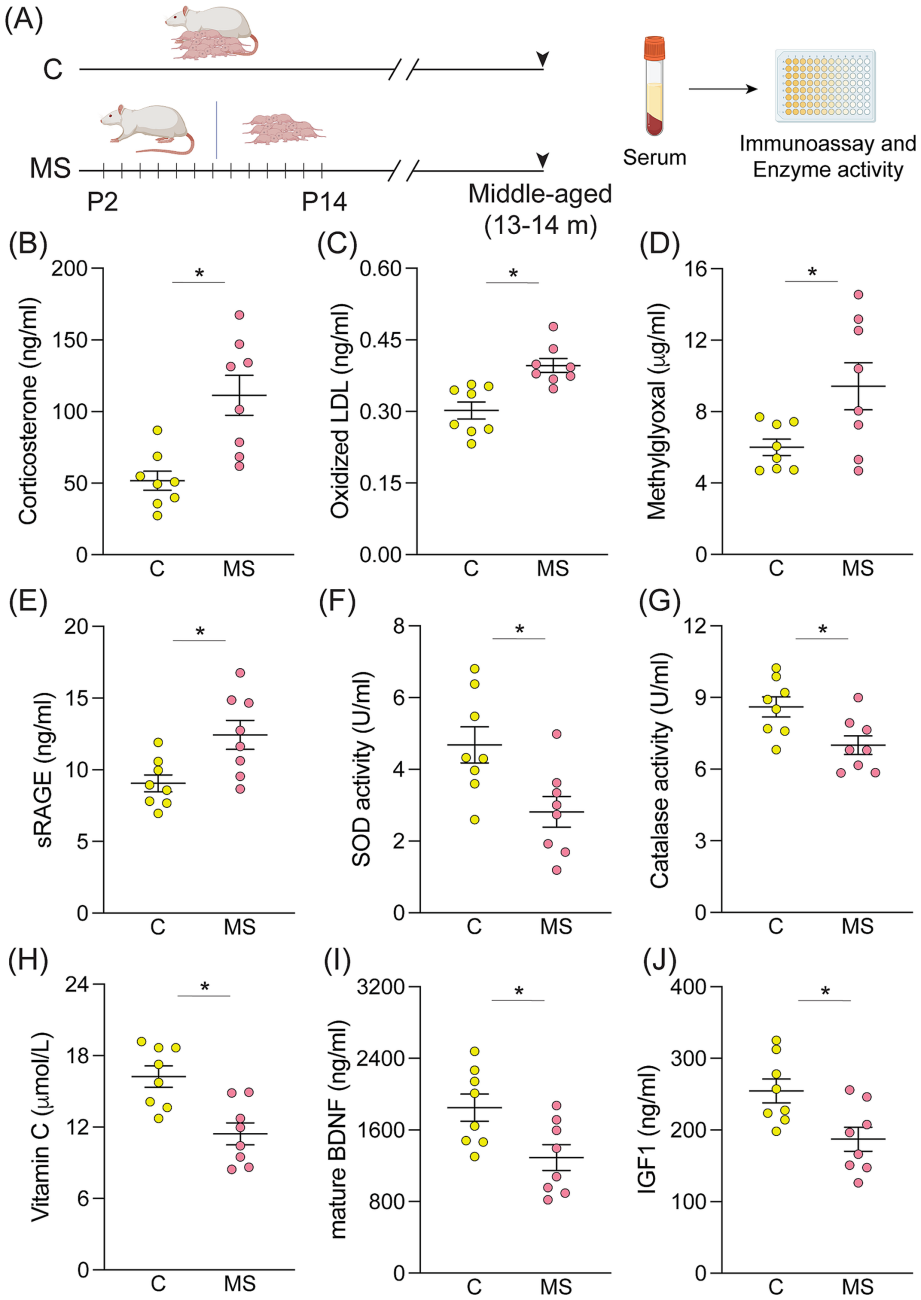
Middle-aged rats with a history of maternal separation exhibit enhanced systemic inflammation and oxidative stress markers. **(A)** Shown is a schematic representation of the maternal separation (MS) paradigm with serum harvested from middle-aged Control (C) and MS animals for analysis of markers associated with stress response, inflammation, oxidative defense, metabolic regulation, and neurotrophic signaling using immunoassays. Shown are the circulating levels of **(B)** corticosterone, **(C)** oxidized low-density lipoprotein (Ox-LDL), **(D)** methylglyoxal, and **(E)** soluble form of receptor for advanced glycation end-products (sRAGE). Graphs represent enzyme activity of **(F)** superoxide dismutase (SOD) and **(G)** catalase, and indicate circulating **(H)** vitamin C levels. Shown are serum levels of **(I)** brain-derived neurotrophic factor (BDNF) and **(J)** insulin-like growth factor-1 (IGF1). All graphs are from middle-aged control **(C)** and MS animals (in both C and MS, *n* = 8 males). Data are represented as mean ± SEM, with individual data points depicted as dots in the graphs. *p < 0.05 as compared to controls; unpaired Student’s *t*-test.

**Figure 2:**
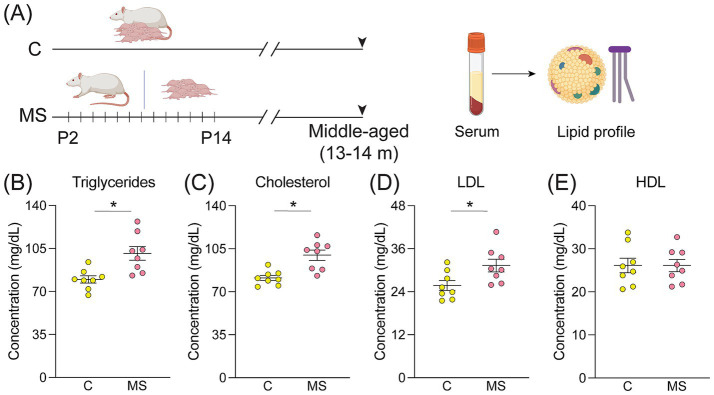
Middle-aged rats with a history of maternal separation exhibit a dysregulated lipid profile. **(A)** Shown is a schematic representation of the maternal separation (MS) paradigm with serum harvested from middle-aged C and MS animals for assessment of the lipid profile. **(B–E)** Shown are circulating levels of **(B)** triglycerides, **(C)** cholesterol, **(D)** low-density lipoprotein (LDL), and **(E)** high-density lipoprotein (HDL) in middle-aged C and MS animals (in both C and MS, *n* = 8 males). Data are represented as mean ± SEM, with individual data points depicted as dots in the graphs. *p < 0.05 as compared to controls; unpaired Student’s *t*-test.

We sought to investigate the influence of MS on several serum markers in middle-aged male rats, wherein we evaluated the circulating levels of peripheral markers associated with stress responsivity (corticosterone and Cort), glyco-oxidative homeostasis [oxidized low-density lipoprotein (Ox-LDL), methylglyoxal (MGO), and soluble form of receptor for AGEs (sRAGE), superoxide dismutase (SOD) and catalase activity, and vitamin C levels], growth factors (BDNF and IGF1; Figure 1A). Middle-aged MS rats exhibited a significant increase in Cort levels when compared to their age-matched controls (Figure 1B). Furthermore, the immunoassays revealed elevated levels of glyco-oxidative stress markers, namely Ox-LDL, MGO, and sRAGE (Figures 1C–E) in MS animals when compared to age-matched controls. Additionally, the enzyme activity assays revealed significant reductions in the activity of the antioxidant enzymes SOD and catalase (Figures 1F,G) concurrent with a decline in serum vitamin C levels, which also serves a potent antioxidant role, in middle-aged MS rats (Figure 1H). We observed a significant reduction in peripheral levels of circulating growth factors, namely BDNF and IGF1, in middle-aged MS animals (Figures 1I,J). Peripheral lipid profiling in a separate cohort of middle-aged control and MS male rats (Figure 2A) indicated a significant increase in triglyceride, total cholesterol, and LDL levels in the MS cohort (Figures 2B–D), with no change observed in HDL levels (Figure 2E). Importantly, these observed effects for the tested serum biomarkers remained statistically significant following FDR correction (Supplementary Table 3), confirming that adjustment for multiple comparisons did not alter the interpretation of these findings. Furthermore, we find a history of MS programs, persistent increases in markers linked to inflammation and oxidative damage, accompanied by dyslipidemia, disruption of key antioxidant and metabolic pathway enzymes, as well as a reduction in protective trophic factor signaling.

### 3.2 Middle-aged MS rats show an increased accumulation of advanced glycation end-products (AGEs) in circulation

**Figure 3:**
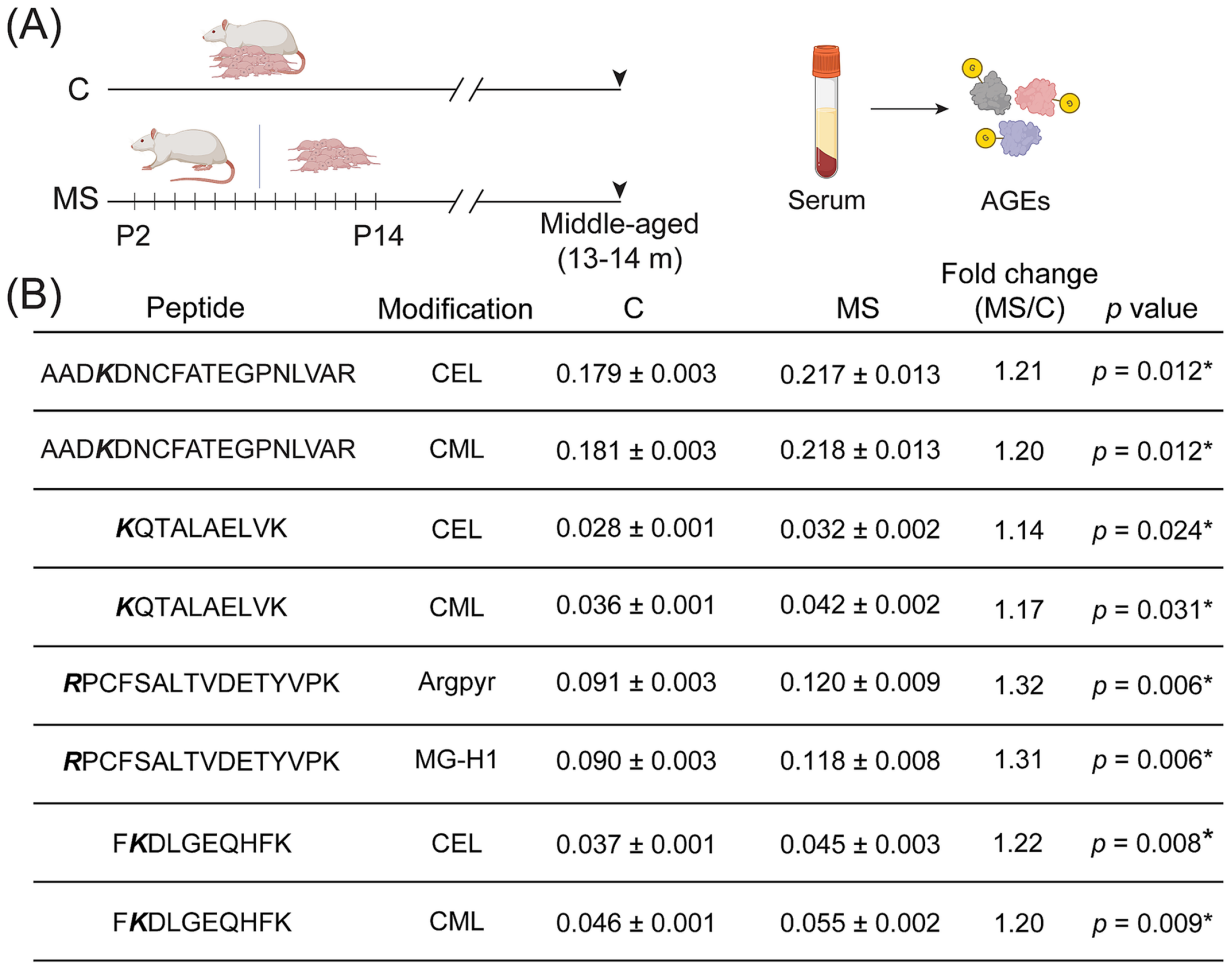
Middle-aged rats with a history of maternal separation exhibit enhanced serum levels of advanced glycation end-products. **(A)** Shown is a schematic representation of the maternal separation (MS) paradigm with serum harvested from middle-aged C and MS animals for assessment of AGEs using SWATH-mass spectrometry analyses. **(B)** Shown are glycated modifications, namely N_ɛ_-carboxymethyl-lysine (CML), N_ɛ_-carboxyethyl-lysine (CEL) on albumin peptides AAD ***K*** DNCFATEGPNLVAR, ***K*** QTALAELVK, F ***K*** DLGEQHFK, and Argpyrimidine (Argpyr), methylglyoxal-derived hydroimidazolone isomer 1 (MG-H1) adducts on albumin peptide ***R*** PCFSALTVDETYVPK, detected using SWATH-mass spectrometry analyses of serum harvested from middle-aged C and MS animals (*n* = 3 per group; each *n* pooled from three rats). The bold and italicized letter in the peptide sequence denotes the specific amino acid with a glycation modification. Data are represented as a ratio of modified to unmodified peptide (mean ± SEM) along with relative fold change as compared to middle-aged C. *p < 0.05 as compared to controls; unpaired Student’s *t*-test.

Given the evidence of enhanced systemic glyco-oxidative damage in middle-aged MS animals, with an increase in circulating MGO, a highly reactive carbonyl species that is a key precursor for advanced glycation end-products (AGEs), we next sought to address the impact of MS on AGEs on serum albumin peptides using a quantitative proteomic technique, namely the SWATH–mass spectra analyses. Serum albumin is a major target for glycation, with progressive accumulation of AGEs contributing to age-associated pathology. In particular, we measured the accumulation of predominant advanced glycation modifications, namely N_ɛ_-carboxymethyl-lysine (CML), N_ɛ_-carboxyethyl-lysine (CEL), Argpyrimidine (Argpyr), and methylglyoxal-derived hydroimidazolone isomer 1 (MG-H1) in serum harvested from middle-aged control and MS male rats (Figure 3A). MS animals exhibited significantly increased glycation on four glycated serum albumin peptides (Figure 3B). We found significantly elevated levels of CML (Supplementary Figure 1) and CEL modifications on albumin peptides, namely AAD*K*DNCFATEGPNLVAR, *K*QTALAELVK, and F*K*DLGEQHFK, whereas increased Argpyrimidine (Argpyr) and MG-H1 modifications were observed on the *R*PCFSALTVDETYVPK serum albumin peptide in MS animals when compared with the controls (Figure 3B). These findings indicate that the early stress of MS is associated with increased systemic accumulation of AGE signatures on serum albumin in middle-aged life, reflective of accelerated signatures of glyco-oxidative stress in animals with a MS history.

### 3.3 Middle-aged MS rats exhibit impaired object recognition memory

**Figure 4:**
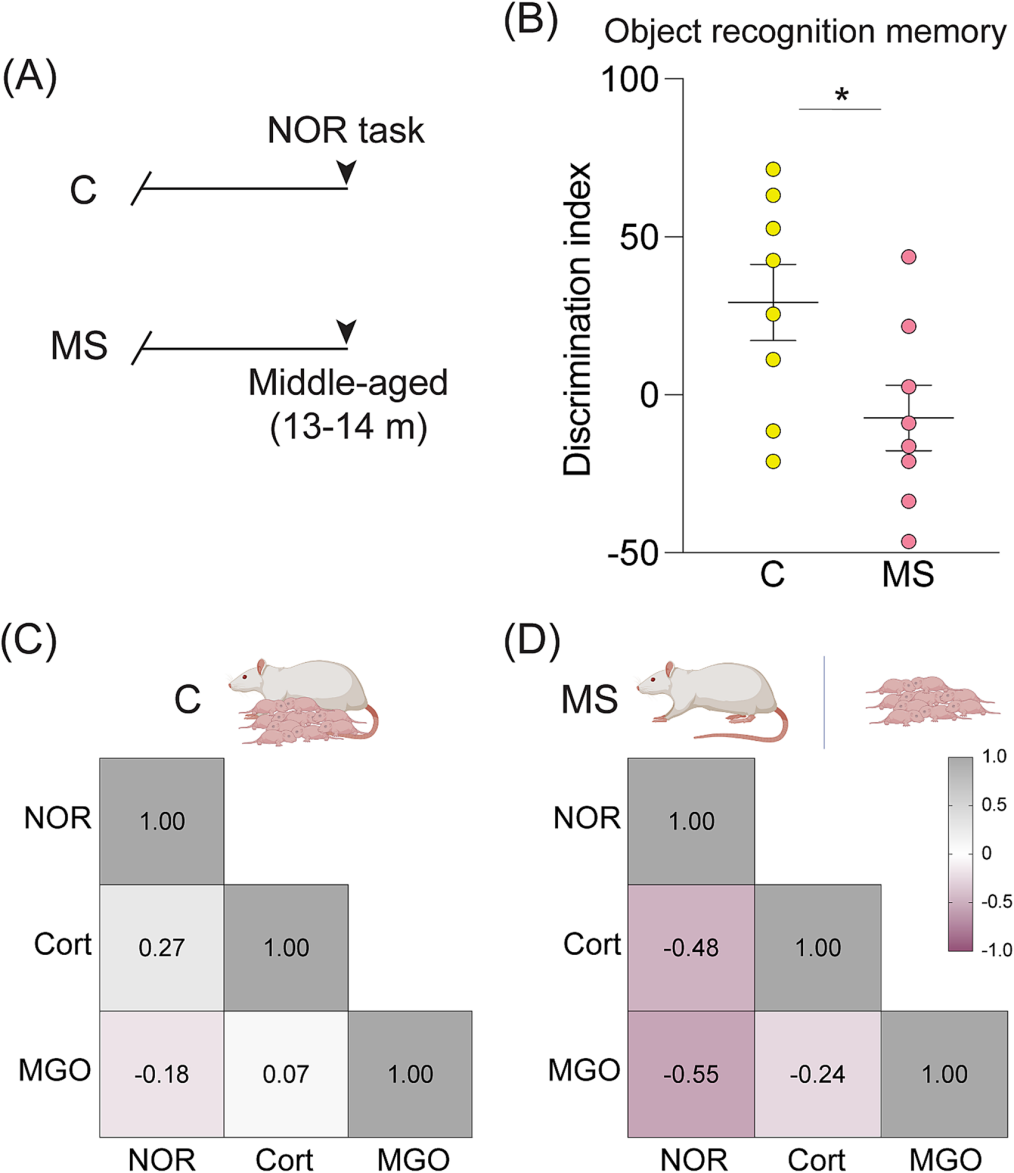
A history of maternal separation drives object recognition memory impairments in the middle-aged rats. **(A)** Shown is a schematic representation of the maternal separation (MS) paradigm, wherein animals from control and MS groups were tested for object recognition memory using the NOR task, with serum harvested post-NOR for analysis of peripheral markers using immunoassays. **(B)** The graph depicts discrimination indices in the middle-aged male rats from both the control and MS groups (in both C and MS, *n* = 8 males). **(C,D)** Shown is a correlation matrix denoting the Pearson’s correlation coefficient (*r*) evaluated between the discrimination index scored using the NOR task and levels of circulating markers, namely Cort and MGO, in middle-aged **(C)** control and **(D)** MS animals (in both C and MS, *n* = 8 males). The underlying matrix indicates the nature and extent of association, with positive correlations shown in gray and negative correlations shown in lilac. Data represented as a Pearson’s correlation coefficient ranging from −1 to +1 and derived from a comparison to the NOR score of +1.

We then sought to examine whether middle-aged animals with a MS history exhibit changes in cognitive performance that might accompany the altered peripheral physiological markers by assessing object recognition memory using the NOR task (Figures 4A,B). We noted that the discrimination indices evaluated on the NOR task were significantly lowered in middle-aged MS animals compared to their age-matched controls (Figure 4B). To address whether the extent of cognitive impairment was correlated with changes in serum markers linked to stress and AGEs, we performed the cross-correlation analyses for the discrimination indices using the Cort and MGO in the control and MS middle-aged groups. Although the pattern indicates increased Cort and MGO levels in middle-aged MS animals, this did not exhibit a significant cross-correlation with NOR discrimination indices in the MS or control cohorts (Figures 4C,D).

## 4 Discussion

The findings of our study indicate that the early stress of MS exerts long-lasting consequences on physiology, as evidenced by significant alterations in specific serum glyco-oxidative stress markers, stress responsivity, antioxidant pathways, trophic factor signaling, and accompanied dyslipidemia. Furthermore, MS is accompanied by significant increases in levels of the precursor for AGEs, MGO, an accumulation of AGE modifications on serum albumin, and an increased expression of sRAGE levels. Furthermore, we noted that impaired object recognition memory was scored on the NOR task in middle-aged MS male rats. Collectively, these findings reveal a pattern of the disrupted expression of several markers linked to physiological aging in middle-aged MS male rats. We focused on serum for peripheral profiling because it provides insight into understanding of whole-body status, and opens up the possibility of potential translational biomarkers for early adversity.

Early stress is well known to drive persistent perturbations in stress hormones, in particular by disrupting top-down and regulatory feedback control of the hypothalamic–pituitary–adrenal (HPA) axis (Van Bodegom et al., 2017). Multiple studies with the MS model indicate elevated corticotropin-releasing factor (CRF) expression within the paraventricular neurons of the hypothalamus, which is thought to underlie potentiated HPA axis activity under both baseline and stressful conditions (Plotsky and Meaney, 1993; Plotsky et al., 2005). Furthermore, decreases in glucocorticoid receptors in key regulatory circuits like the hippocampus and prefrontal cortex are linked to impaired negative feedback control over the HPA axis, thus driving disruption of both normal circadian shifts and stress-mediated secretion of corticosterone (Myers et al., 2014; Spencer et al., 2018). Most studies have restricted their analysis to the impact on the HPA axis and serum corticosterone levels until adulthood, with few exceptions that have examined the impact of early stress history as animals age (Vallée et al., 1999; Nishi et al., 2014; Van Bodegom et al., 2017). A prior study with a similar MS model reported enhanced corticosterone levels in four, but not 10-month-old male rats, and no changes in stress reactivity (Ruiz et al., 2018). A study performed with prenatal stress in rats indicated no change in baseline corticosterone, but sustained enhancement following stress, with a slower return to baseline (Vallée et al., 1999). In our results, we find that middle-aged MS rats, sacrificed at time-points when corticosterone levels are expected to be at the trough of the circadian rhythm, have significantly elevated corticosterone levels when compared to their age-matched controls. It would also be of substantial interest to explore whether animals with a history of MS exhibit changes in the magnitude of diurnal Cort fluctuations and HPA axis feedback sensitivity, as they age. This may indeed serve as one of the key factors in animals with an early stress history that, when overlaid on the natural processes of biological aging may hasten age-related physiological and functional decline.

The serum profiling performed in our studies revealed a clear pattern of enhanced oxidative stress, with enhanced expression of Ox-LDL, a decline in serum antioxidant enzyme activity of SOD and catalase, and reduced vitamin C levels. Collectively, these points to a picture wherein MS animals in middle-aged life exhibit a failure of their antioxidant defense pathways, which may predispose them to an enhanced vulnerability for oxidative damage, a key driver of biological aging. Prior studies indicate either no change or a decline in serum SOD activity, along with tissue-specific changes in the expression/activity of distinct antioxidant enzymes during adulthood in different models of early stress (Ho et al., 2016; Réus et al., 2017; Fenton Navarro et al., 2024), suggesting that the severity of the early stressor may determine the time-point and degree of alterations in key pathways that buffer against oxidative damage. Taking into account that reduced serum SOD activity has been documented in individuals with neurological conditions including epilepsy, ischemic stroke, and major depressive disorders (Zhang et al., 2022; Chidambaram et al., 2024), it would be of interest to investigate whether lower circulating SOD activity noted in middle-aged MS animals is associated with similar changes in the brain, and the consequent impact on function. Although the commonly used MS model, characterized with a daily three-hour separation of pups from their dam from P2–P14, is often regarded as a relatively subtle early stressor, the changes observed are profound in their persistence, remaining evident a year after stress cessation and highlighting the long-lasting impact of early stress on outcomes across the life span. In this regard, one can think about biological aging as the ‘second hit’ that unmasks the heightened vulnerability in animal models with early stress history. Another key hallmark of oxidative stress is advanced glycated end-products (AGEs), which are formed through non-enzymatic glycation and oxidation of proteins and lipids, accumulating progressively with age, metabolic stress, and in several pathophysiological contexts. We found that one of the key precursors for AGEs, MGO, is elevated in the serum of middle-aged MS animals. The highly reactive alpha-ketoaldehyde MGO has been implicated in many aging-related disorders, from metabolic dysfunction and cancer to neurodegeneration, and is reported to evoke diverse cytotoxic effects on both the brain parenchyma and neurovasculature (Kold-Christensen and Johannsen, 2020; Berends et al., 2023). For our study, albumin was selected as the target protein for glycation analysis because it is the most abundant serum protein in rats and contains 53 lysine and 24 arginine residues that are susceptible to glycation (Anguizola et al., 2013; Vetter, 2015; Bateman et al., 2025). We observed increased glycation at four distinct albumin residues in MS rats, three of which align with known glycation-prone sites in human serum albumin (Anguizola et al., 2013; Soboleva et al., 2019). Among the significantly enhanced modifications observed on specific serum albumin peptides from middle-aged MS rats was that of the AGE methylglyoxal-derived hydroimidazolone isomer 1, indicating that the elevated serum MGO levels are accompanied by robust MGO-based AGE signatures. Oxidative stress is known to increase MGO production by perturbing glycolysis and depleting glyoxalase-mediated detoxification mechanisms, leading to the accumulation of this cytotoxic metabolite and the subsequent formation of advanced glycation end-products (Maessen et al., 2015). Furthermore, oxidative stress and MGO form a self-perpetuating cycle, establishing a positive feedback loop in which reactive oxygen species impair the glyoxalase system, thereby exacerbating dicarbonyl stress and cellular dysfunction. Consequently, the accumulation of MGO promotes the formation of AGEs, which may further drive oxidative stress through the RAGE axis (Rabbani et al., 2016; Vašková et al., 2025). While our focus was on serum albumin, it is of interest to note that several other proteins are targets for AGEs, spanning from structural proteins, epigenetic writer and eraser enzymes, antioxidant defense pathways, and suggesting the broad impact of impaired glycation on metabolism and physiology. While interpreting our proteomic results, it is important to keep in mind the consideration that we pooled serum samples to generate sufficient experimental material, which carries the inherent risk of missing less abundant signatures in individual samples. In this regard, our proteomic results are exploratory and motivate future experiments to identify the entire gamut of targets for AGE modifications in animals with a history of early stress. The ligand AGEs, through RAGE signaling, can impinge on inflammatory pathways, driving up peripheral and central inflammation that is considered a key driver of aging processes (Vitorakis and Piperi, 2024; Zhang et al., 2025). Although several studies indicate that the soluble form of RAGE (sRAGE) exerts anti-inflammatory effects by functioning as a decoy receptor that promotes AGE clearance from circulation, few reports suggest that sRAGE levels increase as a compensatory protective response to heightened oxidative and inflammatory conditions (Yamagishi et al., 2006; Nakamura et al., 2007; Erusalimsky, 2021; Kaźmierski et al., 2021). Thus, the accumulation of AGEs on serum albumin assessed by the mass spectrometric methods and concomitant increases in serum MGO and sRAGE levels evaluated using immunoassays collectively indicate heightened glyco-oxidative stress in middle-aged animals with an MS history. This is associated with a dysregulated lipid profile, with increased circulating triglyceride, total cholesterol, and LDL levels. In this study, we also noted that enhanced circulating Ox-LDL acts as a key marker of oxidative stress and inflammation in middle-aged MS animals. Given that atherogenic modifications of LDL, such as glycation and desialylation, are commonly detected in the circulation of individuals with atherosclerosis and diabetes (Poznyak et al., 2023), our study motivates future studies to profile AGEs on specific lipid species in middle-aged MS animals to address whether early stress can contribute to increased cardiometabolic risk via glycation of lipids. Collectively, the overlay of dyslipidemia and accumulation of AGEs in circulation could exacerbate inflammatory pathways, potentially influencing biological aging trajectories in animals with a history of early stress.

Along with enhanced markers of oxidative damage and heightened accumulation of AGEs, we also observed a significant decline in protective pathways in middle-aged MS male rats, namely in trophic factor signaling (BDNF and IGF1). BDNF is a key regulator of neural function and plasticity, and its expression is modulated by diverse stressors, including early stress (Nair et al., 2007; Cirulli et al., 2009; Suri et al., 2013; Wang et al., 2022). Several previous studies have shown that the expression of BDNF in limbic brain regions is reduced in animal models of early stress, albeit with these changes dependent on the time-point of assessment, with middle-aged animals showing a decline in hippocampal BDNF expression associated with a reduction in activated histone marks (Suri et al., 2013). Consistent with clinical literature indicating early stress evokes a decrease in serum BDNF levels during adulthood (Kim et al., 2024), we also find that an animal model of early stress is associated with a decline in serum BDNF levels in middle-aged life. Animal models of both pre- and post-natal stress have documented alterations in the IGF1 levels and function, predominantly in adulthood (Basta-Kaim et al., 2014; Ghosh et al., 2016), with our results pointing to the robust persistence of a decline in IGF1 levels in the serum of middle-aged MS animals. These findings reveal a pattern of lowered trophic support already apparent in circulating levels of BDNF and IGF1, likely reflecting a reduction in tissue availability of key trophic factors that play an important role in cell survival and plasticity, and are reported to decline in major depression and neurodegenerative diseases (van Varsseveld et al., 2015; Wrigley et al., 2017; Emon et al., 2020; Numakawa and Kajihara, 2025). Our results reveal coincident changes with increased circulating stress hormones (Cort), enhanced glyco-oxidative markers (AGEs, MGO, Ox-LDL, and sRAGE) combined with a decline in trophic factors (BDNF and IGF1) and oxidative defense modulators (SOD, catalase, and vitamin C) in the periphery, which could contribute to an attenuation of stress-resilience pathways, thus creating ripe conditions to enhance vulnerability for aging processes. Importantly, these findings remained significant even after adjustment for multiple comparisons, supporting the robustness of the observed systemic effects driven by MS history in middle-aged life.

We also conclude that animals with an MS history exhibit cognitive decline in middle-aged life, in agreement with prior reports. While prior preclinical and clinical studies report a role for elevated circulating Cort and MGO levels in driving cognitive impairment (Belanoff et al., 2001; Li et al., 2006; Comijs et al., 2010; Beeri et al., 2011; Pucci et al., 2021), we did not observe any significant correlations between the discrimination indices on the NOR and circulating Cort and MGO levels in Control or MS cohorts. We have not profiled the cross-correlations of BDNF, IGF1, Ox-LDL, and AGEs with cognitive performance, and future experiments are required to closely assess the relationship between these peripheral markers that are altered in MS animals and their contributions to cognitive aging. One of the key challenges with studies on diverse early adversity models is the ability to causally link brain function and behavioral changes to system-wide alterations in signatures of stress responsivity, oxidative metabolism pathways, and trophic factor signaling, with most reports providing evidence that is largely correlational. In this regard, our findings also reveal several peripheral signatures in middle-aged MS animals that may reflect systemic changes with potential impact on brain function and prompt future experiments to directly assess which of these systemic factors contributes to early adversity-driven cognitive decline.

While growing evidence highlights the impact of early-life stress on peripheral signatures of inflammation and oxidative stress (Pedersen et al., 2018; Chen et al., 2021; Chaudhari et al., 2022, 2026; Fenton Navarro et al., 2024), there is a relative paucity of literature beyond the adulthood timepoint, examining the levels of these markers at later time-points in life, which provides valuable insight into the temporal emergence of maladaptive peripheral alterations. Furthermore, several studies suggest that early-life adversity may hasten biological aging processes (Drury et al., 2012; Suri et al., 2013; Epel and Prather, 2018; Chaudhari et al., 2022, 2026; Künzi et al., 2024; Sapolsky, 2024), although our present findings are restricted to a single time-point in middle-aged life, making it difficult to comment on the acceleration of aging processes in the absence of multiple chronological readouts. Longitudinal studies that span across multiple epochs of the life-span will be required to assess whether there are indeed possible inflection points when physiological aging processes are hastened in animals with a history of MS. A point to note is that the choice of control cohorts used in our present study was animal facility reared control litters and dams that were left undisturbed in their home cages across the life-span, except for routine animal facility procedures. To delineate consequences of MS, diverse comparative groups have been used as controls in the literature, which includes animal facility reared and brief postnatal handling (3–15 min daily separation from dams) cohorts (Bhatnagar and Meaney, 1995; Lehmann et al., 2002; Korosi and Baram, 2009; Hendricks et al., 2012; Suri et al., 2013; Endo et al., 2021). We used the age-matched animal facility reared cohort for our controls, given postnatal handling itself has an impact on a diversity of measures including adult HPA-axis reactivity promoting stress resilience effects and cognitive function (Meaney et al., 1991; Bhatnagar and Meaney, 1995; Vallée et al., 1999; Lehmann et al., 2002; Fenoglio et al., 2005; Kosten et al., 2007; Macrì et al., 2008; Korosi and Baram, 2009) and our results should be interpreted keeping in mind the choice of control cohort when comparing across studies. Specific caveats to consider when interpreting the results of our brief report are that, although individual animals in experimental groups were derived from 3–4 litters to minimize litter effects, litter origins were not tracked across the lifespan into middle-aged life. Hence, we cannot completely rule out the possibility of potential litter of origin contributions to our readouts. Future studies are needed with larger experimental cohorts to more extensively assess the biomarkers tested in our brief report, including tracking of litter origin across the life span and inclusion of female rats to evaluate potential sex-dependent effects.

In summary, our findings reveal that the early stress of MS programs long-lasting changes in the expression of several serum markers, indicating dyslipidemia, perturbed oxidative damage, altered trophic signaling, and increased AGEs, in addition to significant cognitive decline on the NOR task, which could serve to impact diverse aspects of biological aging. Taken together, this pattern of changes suggests a predisposition toward altered biological aging trajectories in animals with a history of MS. This study motivates future clinical studies to assess the translational importance of these putative biomarkers for age-related dysfunction in individuals with a history of early adversity.

## Data Availability

The datasets presented in this study can be found in online repositories. The names of the repository/repositories and accession number(s) can be found at: https://www.ebi.ac.uk/pride/archive/, PXD074495.
