# Supplementary material for: A history of maternal separation drives systemic aging-associated signatures in middle-aged male rats
Source: Front Cell Neurosci. 2026 May 4;20:1809602. doi: 10.3389/fncel.2026.1809602 (PMC13180572; doi:10.3389/fncel.2026.1809602)
Supplement: Supplementary file 1 [file Data_Sheet_1.pdf]

Supplementary Table 1: Details of precursor and fragment ions used for the quantification of glycated peptides of rat serum albumin in middle-aged life

Peptide	Precursor ion m/z	Precursor ion charge state	Fragment ions used for precursor ion quantification (m/z)
AADKDNCFATEGPNLVAR	650.3109	3	$b7^{+}$(775.3039), $b9^{+}$(993.4095), $y6^{+}$(669.4042), $y7^{+}$(726.4257), $y8^{+}$(855.4683), $y9^{+}$(956.5160), $y10^{+}$(1027.5531)
AAD***K(CML)***DNCFATEGPNLVAR	669.6461	3	$b11^{+2}$(641.2562), $y4^{+}$(458.3085), $y5^{+}$(572.3515), $y7^{+}$(726.4257), $y8^{+}$(855.4683), $y9^{+}$(956.5160), $y10^{+}$(1027.5531)
AAD***K(CEL)***DNCFATEGPNLVAR	674.3179	3	$y7^{+2}$(363.7165), $y4^{+}$(458.3085), $y5^{+}$(572.3515), $y7^{+}$(726.4257), $y8^{+}$(855.4683), $y9^{+}$(956.5160), $y10^{+}$(1027.5531)
KQTALAELVK	550.8373	2	$b5^{+}$(542.3297), $b7^{+}$(742.4094), $b8^{+}$(855.4934), $b9^{+}$(954.5619), $y7^{+}$(743.4662), $y8^{+}$(844.5138), $y9^{+}$(972.5724)
K(CML)QTALAELVK	579.8401	2	$b8^{+}$(913.4989), $y3^{+}$(359.2653), $y5^{+}$(559.3450), $y6^{+}$(672.4291), $y7^{+}$(743.4662), $y8^{+}$(844.5138), $y9^{+}$(972.5724)
K(CEL)QTALAELVK	586.8479	2	$b6^{+}$(685.3879), $y3^{+}$(359.2653), $y5^{+}$(559.3450), $y6^{+}$(672.4291), $y7^{+}$(743.4662), $y8^{+}$(844.5138), $y9^{+}$(972.5724)
FKDLGEQHFK	416.8839	3	$b3^{+}$(391.1976), $y8^{+2}$(487.2405), $y9^{+2}$(551.2880), $y3^{+}$(431.2401), $y4^{+}$(559.2987), $y6^{+}$(745.3628)
F***K(CML)***DLGEQHFK	436.2191	3	$y6^{+2}$(373.1850), $y8^{+2}$(487.2405), $y9^{+2}$(580.2907), $y4^{+}$(559.2987), $y5^{+}$(688.3413), $y6^{+}$(745.3628)
F***K(CEL)***DLGEQHFK	440.8909	3	$y6^{+2}$(373.1850), $y8^{+2}$(487.2405), $y4^{+}$(559.2987), $y5^{+}$(688.3413), $y6^{+}$(745.3628)
RPCFSALTVDETYVPK	628.3171	3	$b6^{+}$(719.3294), $b7^{+}$(832.4134), $b8^{+}$(933.4611), $b8^{++}$(467.2342), $y7^{+}$(851.4145), $y8^{+}$(950.4829), $y9^{+}$(1051.5306)
R(Argpyrimidine)PCFSALTVDETYVPK	654.9925	3	$b6^{+}$(799.3555), $y4^{+}$(506.2973), $y5^{+}$(607.3450), $y6^{+}$(736.3876), $y7^{+}$(851.4145), $y8^{+}$(950.4829), $y9^{+}$(1051.5306)
R(MG-H1)PCFSALTVDETYVPK	646.3206	3	$b5^{+}$(702.3028), $y4^{+}$(506.2973), $y5^{+}$(607.3450), $y6^{+}$(736.3876), $y7^{+}$(851.4145), $y8^{+}$(950.4829), $y9^{+}$(1051.5306)
