# Supplementary material for: A history of maternal separation drives systemic aging-associated signatures in middle-aged male rats
Source: Front Cell Neurosci. 2026 May 4;20:1809602. doi: 10.3389/fncel.2026.1809602 (PMC13180572; doi:10.3389/fncel.2026.1809602)
Supplement: Supplementary file 2 [file Data_Sheet_2.pdf]

Supplementary Table 2: Area under the curve for unmodified and glycated albumin peptides from serum harvested in middle-aged control and MS rats

Peptide	ModifiCation	Area under the curve																	
		Control1_1	Control1_2	Control1_3	Control2_1	Control2_2	Control2_3	Control3_1	Control3_2	Control3_3	MS1_1	MS1_2	MS1_3	MS2_1	MS2_2	MS2_3	MS3_1	MS3_2	MS3_3
AADKDNCFATEGPNLVAR	Unmodified	19217832	19244710	17102276	24481068	25294456	25669602	25553156	24798650	24612738	19556880	20117742	19595468	22742920	23175076	24128532	18875822	18577994	16138912
	CEL	3717183	3441825	3270930	4572163	4507379	4646602	4378897	4303534	4008715	3939273	4128842	3733722	4231666	4255058	4460656	4999073	4964624	4384484
	CML	3716768	3458706	3247233	4614022	4564130	4667374	4401565	4329448	4036320	3992534	4158858	3757777	4213408	4266301	4454028	5056461	4958590	4374075
KQTALAELVK	Unmodified	2073234	2013691	1998915	2772746	2676569	2971179	2767364	2983690	2925710	2338576	2247050	2179869	2782646	2729292	2892304	2194079	2074389	2071240
	CEL	60295	58340	59153	74144	78521	81006	74166	76114	76720	76423	72992	69652	72925	70996	76409	86784	78549	77348
	CML	78735	75161	77816	95648	101524	101588	96243	95550	96791	100115	99450	90937	95148	87338	93681	110004	106405	97335
RPCFSALTVDETYVPK	Unmodified	5908664	5630941	5556759	6386880	6260973	6944200	7229834	7639230	7107319	5507695	5149481	5171539	6719313	7154311	6860271	5582353	6717183	6119559
	Argpyrimidine	582778	570142	579313	597457	613466	568997	584274	618535	587382	575247	652837	573710	694307	633176	644075	876772	1002612	896464
	MG-H1	574401	547736	572565	611249	599065	549077	563435	601774	600106	568319	638045	561656	682697	619192	653550	847592	986746	882011
FKDLGEQHFK	Unmodified	2266811	1968330	1826873	3741249	3593285	3302523	3181169	2825363	2958589	2534587	2227177	2228575	3025163	2783669	2863395	2176568	1921762	1822140
	CEL	81964	78432	78047	132628	129302	124638	114533	104025	106089	110368	93348	95008	118207	105193	111933	121075	97062	106538
	CML	113343	97300	96968	169213	161345	145286	142846	123586	126356	135895	119185	114032	144539	131675	135041	139792	115921	121153

MS	Maternal separation
CEL	Nε-carboxyethyl-lysine
CML	Nε-carboxymethyl-lysine
MG-H1	Methylglyoxal-derived hydroimidazolone isomer 1
