# Supplementary material for: A history of maternal separation drives systemic aging-associated signatures in middle-aged male rats
Source: Front Cell Neurosci. 2026 May 4;20:1809602. doi: 10.3389/fncel.2026.1809602 (PMC13180572; doi:10.3389/fncel.2026.1809602)
Supplement: Supplementary file 3 [file Data_Sheet_3.pdf]

Supplementary Figure 1

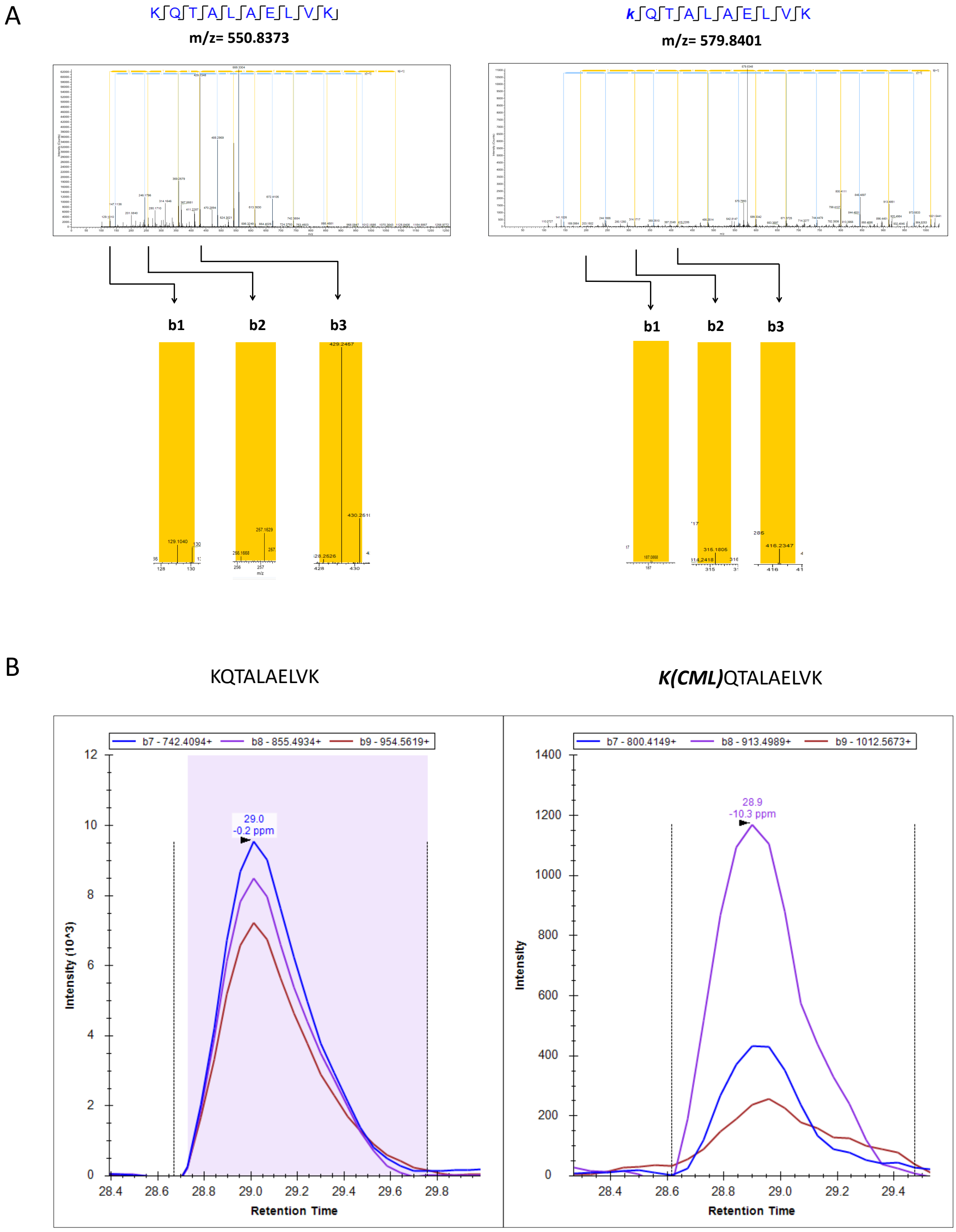

Supplementary Fig 1. (A) Representative MS/MS annotation of CML modified peptide KQTALAELVK with the respective b ions of unmodified peptide KQTALAELVK showing a mass difference of 58 Da, confirming the presence of the CML modification on serum albumin peptide. **(B)** Representative b ion peaks of unmodified and CML-modified peptide KQTALAELVK obtained by Skyline software with the respective b ions having a mass difference of 58 Da, corresponding to the CML modification on serum albumin peptide.
