# Supplementary material for: A history of maternal separation drives systemic aging-associated signatures in middle-aged male rats
Source: Front Cell Neurosci. 2026 May 4;20:1809602. doi: 10.3389/fncel.2026.1809602 (PMC13180572; doi:10.3389/fncel.2026.1809602)
Supplement: Supplementary file 4 [file Data_Sheet_4.pdf]

Supplementary table 3: Statistical analyses

Figure	Readout	Data structure	Statistical test	Statistical analysis	Post-FDR correction
Fig.1B	Corticosterone	Normal	unpaired Student’s *t*-test	$p = 0.004$	$p_{FDR} = 0.014$
Fig. 1C	Oxidized LDL	Normal	unpaired Student’s *t*-test	$p = 0.001$	$p_{FDR} = 0.009$
Fig. 1D	Methylglyoxal	Normal	unpaired Student’s *t*-test	$p = 0.026$	$p_{FDR} = 0.027$
Fig. 1E	sRAGE	Normal	unpaired Student’s *t*-test	$p = 0.011$	$p_{FDR} = 0.023$
Fig.1F	SOD activity	Normal	unpaired Student’s *t*-test	$p = 0.028$	$p_{FDR} = 0.028$
Fig. 1G	Catalase activity	Normal	unpaired Student’s *t*-test	$p = 0.014$	$p_{FDR} = 0.023$
Fig. 1H	Vitamin C	Normal	unpaired Student’s *t*-test	$p = 0.002$	$p_{FDR} = 0.010$
Fig. 1I	Mature BDNF	Normal	unpaired Student’s *t*-test	$p = 0.018$	$p_{FDR} = 0.023$
Fig. 1J	IGF1	Normal	unpaired Student’s *t*-test	$p = 0.018$	$p_{FDR} = 0.023$
Fig.2B	Triglycerides	Normal	unpaired Student’s *t*-test	$p = 0.004$	$p_{FDR} = 0.009$
Fig. 2C	Cholesterol	Normal	unpaired Student’s *t*-test	$p = 0.001$	$p_{FDR} = 0.005$
Fig. 2D	LDL	Normal	unpaired Student’s *t*-test	$p = 0.025$	$p_{FDR} = 0.033$
Fig. 2E	HDL	Normal	unpaired Student’s *t*-test	$p = 0.995$	$p_{FDR} = 0.995$
Fig. 4B	NOR task	Normal	unpaired Student’s *t*-test	$p = 0.038$	*Not applicable*

Fig. 4C: *p values* for correlational data (Control animals)

	NOR	Cort	MGO
NOR		0.516	0.676
Cort	0.516		0.878
MGO	0.676	0.878	

Fig. 4D: *p values* for correlational data (MS animals)

	NOR	Cort	MGO
NOR		0.231	0.158
Cort	0.231		0.563
MGO	0.158	0.563	
